# Improving Follow-Up Skeletal Survey Completion in Children with Suspected Nonaccidental Trauma

**DOI:** 10.1097/pq9.0000000000000567

**Published:** 2022-06-14

**Authors:** Iram J. Ashraf, Danielle Faivus Ackley, Kristin Razawich, Ann Botash, Melissa Schafer, Alicia Pekarsky

**Affiliations:** From the SUNY Upstate Medical University Hospital, Department of Pediatrics, Division of Child Abuse Pediatrics, Syracuse New York.

## Abstract

**Introduction:**

The skeletal survey (SS) is used to evaluate and diagnose bone abnormalities, including fractures caused by child abuse. The American Academy of Pediatrics recommends initial SS for all children younger than 24 months old who are suspected victims of abuse and a follow-up skeletal survey (FUSS) 2 weeks later. The latter can further characterize abnormal or equivocal findings, detect ongoing trauma, or fractures too acute for visualization upon initial assessment.

**Methods:**

Preintervention review at our hospital for FUSS completion of children younger than 36 months old yielded a low 40% average monthly completion rate. We reviewed charts of children who underwent SS during the study period for FUSS completion. There were several barriers to FUSS completion, including lack of provider knowledge regarding FUSS importance, lack of an order for FUSS before hospital discharge, absent chart documentation regarding FUSS decision, loss to follow-up, and parental refusal. Interventions targeting the barriers included provider education, protocolizing FUSS scheduling, standardizing documentation, and community pediatrician outreach. The goal was to increase the average monthly FUSS completion rate from 40% to 90% over 1 year.

**Results:**

After interventions implementation, the average monthly FUSS completion rate rapidly increased from 40% to 80%. There was sustained improvement over the subsequent 12 months.

**Conclusions:**

Interventions were implemented sequentially, targeting barriers at various levels of workflow. Provider education was key and helped increase the reliability of intervention implementation. The most effective intervention was protocol change. This approach led to significant improvement in FUSS completion and sustained improvement.

## INTRODUCTION

The skeletal survey (SS) is used in the evaluation and diagnosis of bone disease and other abnormalities, including in children who are suspected victims of physical abuse.^1–15^ SSs are used to look for occult fractures, especially in young children in whom bony injuries may not be apparent based on history or physical exam.^1–15^ Completion of a second SS 2 weeks after the initial study is the recognized standard of care.^1,2,4,7,8,13,16–20^ The follow-up skeletal survey (FUSS) should be performed even if the initial survey is normal. It may detect fractures too acute to visualize on the initial exam, or fractures missed on the initial study. It may also identify ongoing trauma and provide further information regarding abnormal or equivocal findings detected on the initial survey.^1,2,4,7,8,13,16–20^ A FUSS may also provide information useful for the dating of fractures.^1^ Both initial SS and FUSS provide essential information for determining the perceived likelihood of abuse. Children who have an initial SS may not receive a FUSS because: (1) it is not ordered (eg, the provider is not aware that FUSS is needed, or the provider incorrectly deems it unnecessary); and (2) parental refusal. FUSS noncompletion can result in missed abusive injuries. Studies have shown that the FUSS may identify new information that can change the ultimate determination of possible abuse.^1,2,16–19^ Because missed child abuse can lead to further and escalating abuse and even death, FUSS completion is imperative in most cases.^21,22^ This quality improvement study examined the rate of FUSS completion, identified barriers to noncompletion, and created interventions to address those barriers. The project aim was to improve adherence to FUSS guidelines and the FUSS completion rate to prevent missed cases of abuse.

Our hospital nonaccidental trauma (NAT) protocol includes FUSS completion per recommended guidelines set forth by the AAP^1^ but is expanded to include SS and FUSS for all children younger than 36 months of age undergoing a nonaccidental trauma (NAT) evaluation, rather than only those younger than 24 months of age. We perform SS and FUSS in children up to 36 months of age, as there is evidence that doing so extends prevention of missed cases of abuse to this older cohort.^23^ We had clinical concerns because 60% of children who underwent initial NAT evaluation did not receive a FUSS, indicating our hospital NAT protocol was not being followed. This quality improvement (QI) initiative was created to evaluate and address the problem of FUSS noncompletion and to investigate whether targeted interventions would improve compliance with the NAT protocol.

We believe a 40% average monthly FUSS completion rate is too low and risks missing possible physical abuse.^1,2,16–19,21,22^ Our aim was to increase the average monthly percentage of children younger than 36 months of age who received a FUSS from 40% to 90%. Our interventions began in July of 2018 with a goal to achieve 90% FUSS completion by May 31, 2019.

## METHODS

This QI project took place in a 72-bed children’s hospital within an academic medical center that serves a 19-county catchment area spread over a large geographic area of Central and Upstate NY. The hospital is a level 1 Pediatric Trauma Center. It is a comprehensive regional resource and tertiary care facility capable of providing total care for every aspect of injury from prevention through rehabilitation. Care is provided to abused children using collaborative teams, including the Pediatric Trauma Surgery (PTS) team and the Child Abuse Pediatrics (CAP) team. During this study, children requiring admission for NAT were admitted to the PTS team, who managed their evaluations, with input from the CAP team. Follow-up after discharge occurred with a CAP provider or with PTS. Additional relevant providers (ie, who provide care for children suspected of being abused or who can order SSs) include inpatient pediatric, critical care and emergency medicine residents, fellows, attending physicians, and nurse practitioners. Further, adequate performance of a SS and FUSS requires pediatric radiology techniques and expertise not available at any other center in the catchment area.

We followed SQUIRE guidelines in the report out of this study.^24^ We formed a QI team that included child abuse pediatricians, pediatric trauma surgeons, and pediatric radiology members. A preintervention group was created by reviewing charts of children under 36 months old who underwent an initial SS for suspected physical abuse looking for FUSS completion, ordering team, and reason for FUSS noncompletion. Data were displayed on a standard process control U chart. Standard process control chart rules (Shewhart rules) were used to determine centerline shifts. The preintervention group included all children suspected of being abused and who underwent NAT evaluation at our hospital from June 2017 to June 2018. We included all children who received an initial SS in any clinical setting at our institution (ie, ED, urgent care, inpatient, all ambulatory clinics). Not all children who underwent an initial SS required a FUSS for various reasons (eg, they were deemed a victim of an accidental injury or medical mimic of abuse). We excluded cases if there was documentation in the chart that FUSS was not indicated. Although recommended depending on clinical indications, our institutional protocol does not mandate FUSS for siblings of suspected index cases of NAT. We also excluded FUSS completion for siblings of index cases who underwent NAT evaluation (n = 3 in the background chart review and n = 8 in the improvement cycle chart review). Teams who ordered the initial SS were responsible for scheduling the FUSS and following up on the results.

The preintervention group results were discussed with the key multidisciplinary team members. From this discussion, the authors identified barrier themes to FUSS completion. Barrier themes included a lack of knowledge among relevant providers regarding the importance and indications of FUSS, lack of a FUSS order at the time of discharge when one was indicated, lack of chart documentation regarding FUSS decision, lost to follow-up, unacceptable FUSS if the study was done too late or was inadequate, and parental refusal.

We implemented multifactorial, targeted interventions to reduce barriers at various levels of workflow. The team provided FUSS education electronically or through in-person lectures to pediatricians and pediatrics residents, surgery residents, and emergency medicine residents regarding the importance of FUSS and its completion and the need for appropriate documentation of FUSS decisions in the patient chart. The process of scheduling FUSS before discharge was formally included in the hospital NAT protocol. The pediatric radiology team ensured consistency for all FUSS including study timing, study quality, and that FUSS studies were read only by pediatric radiologists. The team created standardized documentation templates in our electronic medical record system (EMR) to facilitate FUSS decision-making with the aid of an electronic child abuse rating scale.^25^ The scale was used as an adjunct to the provider’s opinion regarding the likelihood of abuse. It also served as a communication tool to help explain the degree of concern for abuse. The rating scale is 7 points denoting levels of concern for abuse ranging from “definitely not inflicted injury” at number 1 to “definite inflicted injury” at number 7. With each level, there are example injuries and clinical scenarios provided to aid decision-making. Any level of concern of ≥3 required a FUSS (in addition to the rest of the NAT evaluation). A FUSS was not required for children with no concern for abuse. The child’s PCP was engaged using an electronic letter template designed to notify them of their patient’s NAT evaluation, decision for FUSS exam, and provide background educational information regarding the FUSS. As interventions were implemented the improvement team regularly reviewed data. Cases with new findings on FUSS and FUSS noncompletions were reviewed by child abuse pediatricians to ensure the child’s safety within the home environment. Multidisciplinary discussions throughout the project were helpful to ensure all parties were comfortable that SSs were only done when indicated and would not be canceled without team discussion.

All children who undergo an initial SS for suspected NAT are reported to the New York State Central Register for suspected maltreatment per our hospital protocol. Per mandated reporter guidelines, if a case is already open for investigation due to initial suspicion of abuse, healing findings identified only on FUSS do not require a rereport to investigative authorities. However, all newly identified findings from FUSS are discussed with pertinent medical and investigative teams. Any new injuries potentially representing new incidents of abuse after the initial SS is done, are reported to the Central Register as a new report. FUSS results with equivocal findings or normal variants are also discussed with pertinent teams because the likelihood of abuse may be less.

## RESULTS

**Fig. 1.**
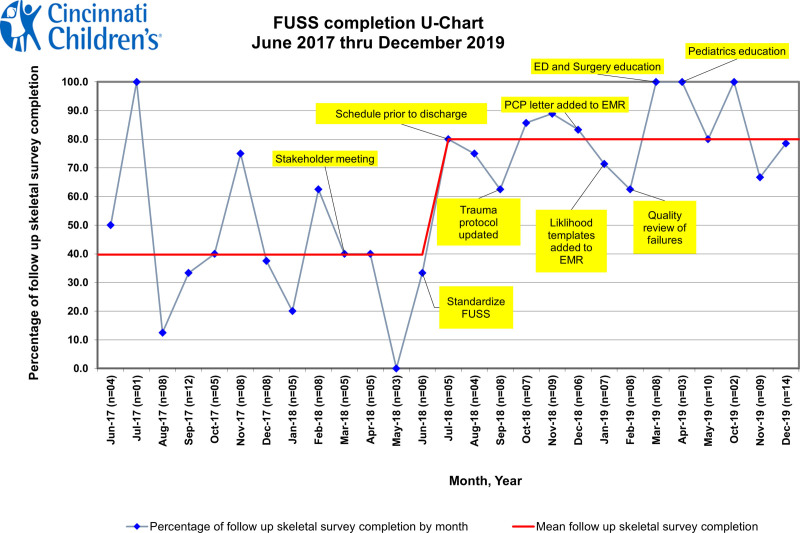
Annotated FUSS Completion U Chart: FUSS adherence rate per month displayed chronologically on a statistical process control chart starting with the preintervention group (June 2017–June 2018), moving to the intervention group (July 2018–May 2019), and ending with the postintervention assessment (October 2019–December 2019). The chart illustrates highest rate of improvement after FUSS standardization and scheduling the FUSS before hospital discharge. It also demonstrates sustained improvement of FUSS completion during the postintervention period.

The preintervention chart review found 78 index children younger than 36 months old who underwent an initial SS for suspected physical abuse. Only 40% (average monthly centerline rate) had a completed FUSS. In addition to the 78 index children who underwent initial SS, there were 3 siblings of index cases and 7 children with documentation in their chart that a FUSS was not required. Results of the preintervention group chart review (June 2017–June 2018) were plotted by FUSS adherence rate per month chronologically on a statistical process control chart (Fig. 1). We used the chart template with permission from Cincinnati Children’s Hospital Medical Center.

**Fig. 2.**
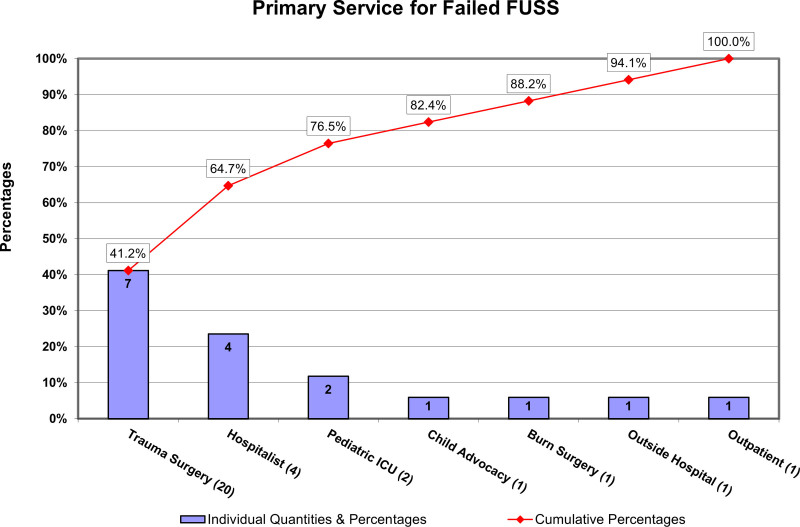
Primary Service for Failed FUSS. Pareto chart illustrates preintervention group FUSS failures by primary service. Pediatric Trauma Surgery service represented the greatest opportunity population.

A sample review of preintervention background charts revealed FUSS failures by primary service, with patients admitted to the PTS team representing the greatest opportunity, as seen in the Pareto chart (Fig. 2).

The average monthly FUSS completion rate increased from 40% to 80% as interventions were implemented. This was sustained over the subsequent 12 months. During the intervention study period (July 2018–May 2019), 75 children underwent initial SS. Sixty of those children completed a FUSS, with a monthly average completion rate of 80% (Fig. 1).

There were no cases of FUSS noncompletion by the PTS team after September 2018, just 3 months after implementing interventions. Noncompletion by teams other than PTS was less frequently seen but required more effort to address. These other FUSS noncompletions were referred to the Institutional Quality Office for review by the department in which they were missed. Improved adherence to FUSS completion was seen thereafter (Fig. 1).

We found that 21.7% (13/60) of children with FUSS during the study period had new findings not seen on the initial SS. Eight (61.5%) of the children with new findings had new injuries on FUSS. Five (38.5%) of the children had new findings that proved initial SS findings were more benign (ie, clarified equivocal findings or proved to be normal variants). Detailed chart review indicated that all patients with new findings on FUSS were highly suspected of abuse even before the FUSS exam.

Reasons for FUSS noncompletion (n = 15, 20% after July 2018) included parental refusal, no-show, or child still hospitalized and too unstable to undergo FUSS. Quality review of 11 cases of noncompletion for reasons initially unknown, revealed provider lack of knowledge regarding FUSS or hospital system challenges in obtaining a FUSS, for why FUSS was not done when indicated.

In the Fall of 2019, we reviewed 22 charts to ensure the positive change was sustained. The FUSS completion rate was 73% (16 of 22 charts), reflecting a sustained practice change (Fig. 1).

## DISCUSSION

The importance of FUSS completion and the potential negative repercussions of noncompletion (ie, high risk of escalating abuse and possible death) made this project a top priority for all key team members in our hospital. The authors identified several barriers to FUSS completion through multidisciplinary meetings and developed targeted interventions to eliminate those barriers.

Updating the NAT protocol to include FUSS scheduling before discharge ensured a mutually agreeable appointment time for both the radiology team and family. Scheduling the FUSS before discharge emphasized provider decision-making regarding its necessity and, in turn, increased documentation of the decision-making using the described EMR templates. The entire process as outlined provided an opportunity to counsel the caregiver regarding the importance of FUSS, and its completion whereas still in the hospital. Incorporating FUSS scheduling into the NAT protocol was a highly reliable intervention resulting in zero noncompletions by the PTS team (Fig. 2).

We involved PCPs as they have presumed established rapport with the family, and it is essential for transparency among all involved to ensure recommendations are followed. With the successful outreach to and engagement of community pediatric providers, we encouraged continued monitoring of these vulnerable children after hospital discharge by communicating abuse-related concerns. Community providers often replied to letters by phone to discuss the details regarding the care of their patient. We have rarely observed this type of response from community pediatricians previously.

We were able to obtain buy-in from not only community providers but also our pediatric hospital radiologists. Coordination with pediatric radiology was crucial to success.

Our interventions directly increased FUSS completion rates and improved FUSS-related documentation. Lost to follow-up and parental refusal were mitigated by counseling caregivers before patient discharge and involving the child’s PCP. We believe the overarching education intervention for hospital providers and PCPs enabled greater success of downstream interventions, ultimately facilitating the increase of FUSS rate completion. Education was directed at increasing provider awareness regarding the utility of FUSS and the importance of case-by-case decision-making regarding its necessity. Clear documentation in the chart of FUSS decision-making was facilitated with the addition of electronic templates that included an abuse likelihood scale to allow communication of abuse concerns.^25^

A similar study was published in 2018 in which investigators attempted to increase their suboptimal rate of FUSS completion in patients for whom it was clinically indicated.^26^ Their intervention was to implement a follow-up clinic for NAT patients. They reported an improvement of 40% to 90% FUSS completion rate. In comparison, our study showed an improvement of 40% to 80%. Our study differed in that loss to follow-up was not our primary barrier, although still a lesser barrier that we addressed. Our primary barrier themes included lack of provider FUSS knowledge and lack of a FUSS order at the time of discharge when one was indicated, among others as discussed. A NAT follow-up clinic is important and ensures completion of all recommended tests related to the NAT evaluation. However, our study also highlighted additional barriers to FUSS noncompletion at multiple levels of workflow, beyond loss to follow-up, which were alleviated with the described interventions. Our barriers may be common to other institutions and as such this work provides examples of successful methods to improve FUSS rates.

The literature suggests that provider discomfort in diagnosing abuse plays a role in FUSS completion and overall NAT management.^27,28^ To alleviate provider variation, provider education, input from a child abuse pediatrician, and protocol implementation are required to reduce biases and reduce cases of missed abuse. Utilizing a protocol for standard of care increases the completion of NAT evaluations^5,29^ and ultimately these interventions increased our FUSS completion rate. Providing electronic templates to input into documentation were also essential in compliance. The interventions are easily reproducible and generalizable. A pediatric trauma center with a child abuse team should be able to evaluate their FUSS completion rate and improve it if it is found to be less than acceptable.

This project’s results emphasize the importance of FUSS exams. Thirteen children (21.7%) who had a FUSS during the study period had new findings not seen on the initial SS. Approximately 62% (n = 8) of children with new FUSS findings had new injuries, whereas 39% (n = 5) had more benign FUSS findings compared with their initial SS. These new injuries found on FUSS emphasized the need for proactive safety planning for the child, including decisions about removing the child from the household. Newly identified findings were discussed with the pertinent authorities who took appropriate next steps in ensuring the victim’s safety.

These results indicate that FUSS detected abusive injuries that may have otherwise been missed. Failure to recognize child abuse can lead to reinjury and death with children being placed back in environments that are high risk for maltreatment.^30^ Compared with children who suffer a single abusive episode, those with recurrent episodes of abuse have higher mortality rates.^31^

Our study has several limitations. First, was the exclusion of FUSS completion for siblings of index cases who underwent NAT evaluation. It is recommended that siblings of index abuse cases be evaluated for abuse with SS if younger than 24 months of age, but FUSS is not required if initial survey is normal.^32^ Since all siblings were excluded from our study, those initial survey findings were not addressed. Second our methodology did not identify all children with suspected physical abuse who were admitted to services other than those included in this study. This limited the ability to fully understand if FUSS completion issues cross all service lines in the same proportion, or if 1 specific team is responsible for an outlying amount of failed FUSS studies. Third, some patients may have been suspected to be abused, but did not have an initial SS, even if one was indicated. Such children were missed in this study, as we were unable to capture them based on our design. Finally, education as an intervention for improvement can have limited sustainability. There must be ongoing provider education, especially when residents, who turnover frequently, are involved in patient care.

Next steps include the continued review of system failures with feedback to primary teams and review of protocols to keep them updated. Ongoing hospital provider education and monitoring of protocol adherence are crucial for sustained improvement. Creating an education program for community pediatricians may further help to sustain acceptable FUSS rates. It is important to note that the standardized evaluation and management of children suspected of being abused reduces the percentage of children who are rehospitalized.^33^

In conclusion, FUSS completion is necessary to detect abusive injuries with the goal of preventing reinjury or death in children who are victims of maltreatment. Our interventions targeted multiple levels of workflow and led to improved FUSS rates. Even though some of the improvement interventions were facilitated using an EMR, they are simple interventions that can be replicated with tailored modification at most, if not all institutions. Improved FUSS completion will decrease missed cases of abuse and the associated morbidity and mortality of this vulnerable population.

## DISCLOSURE

The authors have no financial interest to declare in relation to the content of this article.
